# The Jun N-terminal kinases signaling pathway plays a “seesaw” role in ovarian carcinoma: a molecular aspect

**DOI:** 10.1186/s13048-019-0573-6

**Published:** 2019-10-21

**Authors:** Yingyu Dou, Xiaoyan Jiang, Hui Xie, Junyu He, Songshu Xiao

**Affiliations:** 1 Department of Gynecology and Obstetrics, the third Xiangya Hospital, the Central South University, Changsha, 410013 Hunan China; 2 Cancer Research Institute, the Central South University, Changsha, 410011 Hunan China

**Keywords:** Jun N-terminal kinases pathway, Ovarian cancer, Seesaw role, Anticancer effect, Tumor-promoting effect

## Abstract

Ovarian cancer is the most common gynecological malignancy that causes cancer-related deaths in women today; this being the case, developing an understanding of ovarian cancer has become one of the major driving forces behind cancer research overall. Moreover, such research over the last 20 years has shown that the Jun N-terminal kinase (JNK) signaling pathway plays an important role in regulating cell death, survival, growth and proliferation in the mitogen-activated protein kinases (MAPK) signaling pathway, an important pathway in the formation of cancer. Furthermore, the JNK signaling pathway is often regulated by an abnormal activation in human tumors and is frequently reported in the literature for its effect on the progression of ovarian cancer. Although the FDA has approved some JNK inhibitors for melanoma, the agency has not approved JNK inhibitors for ovarian cancer. However, there are some experimental data on inhibitors and activators of the JNK signaling pathway in ovarian cancer, but related clinical trials need to be further improved. Although the Jun N-terminal kinase (JNK) signaling pathway is implicated in the formation of cancer in general, research has also indicated that it has a role in suppressing cancer as well. Here, we summarize this seemingly contradictory role of the JNK signaling pathway in ovarian cancer, that ‘seesaws’ between promoting and suppressing cancer, as well as summarizing the application of several JNK pathway inhibitors in cancer in general, and ovarian cancer in particular.

## Highlights

The JNK signaling pathway is abnormally activated in patients with ovarian cancer or drug-resistant ovarian cancer.

Autophagy mediated by the JNK signaling pathway plays a dual role in ovarian cancer.

The timing of influencing the JNK signaling pathway will affect the follow-up therapeutic effect.

## Introduction

Ovarian carcinoma (OC) is one of the most common of the gynecologic cancers as well as being the most prevalent cause of gynecology tumor-related deaths worldwide [1]. To date there are some 239,000 new cases and 152,000 deaths due to OC each year [2]. In the United States during 2018 there were about 22,240 new OC cases resulting in 14,070 deaths [3]. Whilst in Europe [1], the OC incidence rate is from 6.0 to 11.4 per 100,000 women, and although it is relatively lower in China, there was at least [4] 52,100 new cases and 22,500 deaths in 2015 alone. Most ovarian carcinomas are diagnosed at an advanced stage, of which 51% are diagnosed at stage III and 29% are diagnosed at stage IV [3, 5] and what are the risk factors for such incidence levels of OC?

Age growth, overweight or obesity, first full-term pregnancy after age 35, fertility therapy, hormone therapy after menopause, family history of OC, breast cancer or colorectal cancer might all be high risk factors for OC [6]. In addition, about 50% of OC patients are more than 65 years old [7] and according to early studies in the Netherlands, patients with stage II and III ovarian cancer, even in the absence of comorbidities, did not achieve the same effective as younger patients [8]. This difference may be related to the relatively poorer physical conditions of the elderly [8]. However, the latest study indicates that older women with OC are 50% less likely to receive standard treatment than younger women, regardless of the type of treatment. Furthermore, when elderly patients receive personalized treatment, it has been shown that the treatment effect on them can be significantly improved [9, 10]. Age itself may not be a high-risk factor [11] and the etiology of OC is unclear but 5–10% of OC is thought to be hereditary. Hereditary OC, like breast cancer, is an autosomal dominant inheritance due to mutations in the BRCA1 and BRCA2 genes. Such gene mutations change the biological effects of cell tissues and, thus, play an indispensable role in promoting the occurrence and development of tumors. According to the dualism of OC, it can be divided into type I ovarian cancer and type II ovarian cancer. Concerning type I OC, the main gene mutations are KRAS, BRAF, PTEN, ARID1A, and PIK3CA, and its onset is slow, the diagnosis is mostly in the early clinical stage, and the prognosis is good. The main mutations in type II OC, however, are TP53 and BRCA1/2 and the onset of the disease is fast, aggressive, no prodromal symptoms, the diagnosis is mostly in the late clinical stage. Ovarian tissue composition is very complex, and it is the organ with the most types of primary tumors of all the organs of the body. There are great differences in different types of histological structure and biological behavior. According to the histological classification of the World Health Organization (WHO) 2014 edition, ovarian tumors can be divided into 14 categories, the main histological types of which are epithelial tumors, germ cell tumors and cord-stromal tumors. Epithelial tumors are the most common histological type of ovarian tumors, and their histology can be further divided into serous, mucinous and endometrioid types. Serous tumors are the main type of ovarian cancer. In addition, the five-year survival rate of serous cancer is 43%, while that of other types of endometrioid cancer is 82%, and that of mucinous cancer is 71% [3].

Although the optimal treatment is surgery as the main component, supplemented with appropriate chemotherapy such as TC (Paclitaxel and carboplatin), TP (Paclitaxe and cisplatin) and PC (cisplatin and cyclophosphamide), in about 70% of patients the ovarian cancer will reoccur. Clinical trials are trying new therapies and drug combinations, such as immunotherapy, target therapy, PARP and anti-angiogenesis drugs. At present, many experiments and clinical trials have been carried out to improve the therapeutic effect on ovarian cancer with the single or combined treatment of potential candidates, and some results have been achieved. However, much remains to be done. Specifically, understanding the genes of abnormally activated or expressed signal transduction pathways in the development of ovarian cancer will be helpful to improve the prognosis of ovarian cancer and the development of new therapies. In this regard, and according to the latest cancer genome research, many signaling pathways have malfunctioned in the development of cancer [12, 13]. For instance, the abnormal activation of the Jun N-terminal kinases signaling pathway, one of the MAPK signaling pathways, has been reported in ovarian cancer frequently, making it one of the most important signaling pathways in the treatment of ovarian cancer [14–17]. To inform our discussions in this paper on the roles of JNK in the death of cells, cancer cell growth, and in chemotherapy resistance, it is worthwhile to first summarize the salient features of both the MAPK and JNK signaling pathways.

## Key features of the MAPK and the JNK signaling pathway

The MAPK pathway includes a series of protein kinases such as ERK1/2, p38 α/β/γ/δMAPK and c-Jun amino terminal kinase 1/2/3(JNK1/2/3), which control cell proliferation, cell survival and cell death in various processes [18, 19]. JNKs, also called ‘stress activated’ protein kinases just like p38 MAPKs, responds to various extracellular stimuli in different organisms.

JNK1/2/3 have been demonstrated in mammals, where splices have revealed more than ten various subtypes of transcription. And the proteins size of JNKs range from fourty-six kDa to fifty-five kDa. JNKs can be activated by cascade phosphorylation reaction of MAPKKK and MAPKK. More than 20 types of MAPKKK have been found such as the MEKK family members protein and the apoptosis related kinases pathway, which regulates and phosphorylates two different types of MAPKK, MKK4/7.MKK4 and MKK7, and activates JNK via phosphorylating the motifs of conserved tripeptide, that is tyrosine 185 and threonine 183 residues [19, 20]. Moreover, scaffold protein, nuclear factor-kB and dual specificity phosphatases also have an effect on the activity of JNKs [19]. Various constitutions of MAPKKK and MAPKK can control JNK signaling. Apoptosis signal regulation kinase 1(ASK1) is in an inactive state in the normal conditions via interacting with thioredoxin [21]. ASK1/MAPKKK5 activating JNK is the mechanism of some kidney diseases [22]. Cypermethrin impairs astrocytes and disrupts the development of the extracellular-matrix via regulating reactive oxygen species (ROS), Ca2+ and JNK pathway [23]. Continuous activation of JNK in hepatocytes can lead to cell death or metabolic abnormalities [24]. TNF related apoptosis- inducing- ligand promotes apoptosis of Bim, homologue of Bcl-2, by activating JNK and its downstream substrates, and enhances hepatocyte cell anti-Fas-induced apoptosis [25]. Increasing ROS inhibits the phosphatase of JNK such as mitogen-activated protein kinase phosphatase 1(MKP1 /DUSP1), which contributes to the continuous activation of JNK [26]. ROS can also remove the inhibition of ASK1 [27]. Furthermore, MAP 2 K/MAP 3 K, interleukin-1, epidermal- growth factor, drugs, endoplasmic reticulum stress (ER stress) and environment stress such as heat, hypoxia as well as radiation can also activate JNK.

There are so many molecular such as STAT1/3, c-jun, c-Myc, FOXO4, Bcl-2, ATF2, Smad2/3, PPARγ1 and RXRα that have been demonstrated as the JNK substrates. JNKs which increase the activity of c-jun transcription via binding and phosphorylating c-jun at Ser73 and Ser63 [28]. C-jun, a part of AP-1 that regulates expression of gene. AP-1, like JNKs can be activated by radiation, various stress or inflammatory cytokine. The activity of c-jun is essential for the Ha-Ras mediating carcinogenesis transformation [29]. Phosphorylated c-jun at Ser73 and Ser63 via Ha-Ras, c-Raf and v-Src induced carcinoma transformation in embryonic fibroblasts [30, 31]. And JNK regulates the activating-transcription factor 2 (ATF-2) and intracellular mitochondrial signaling pathway such as target proteins Sab [32, 33]. Using inhibitory peptide of Tat-Sab interferes with the activity of JNK blocking the increase of ROS.

However, some evidence also shows contradictory phenomena that lacking the JNK1/2 gene induces fetal death in mice. JNKs also control cell autophagy, proliferation or migration. Furthermore, experiments involving the editing of mouse genes suggest that overactivation or loss of the JNK pathway function induces the growth of cancer, inflammation and metabolism related diseases. Some evidence supports the idea that JNK has the function of promoting tumor development, whilst other evidence demonstrate that JNK plays an important role in suppressing cancer. Note that, c-jun is highly expressed in ovarian cancer tissues [34]. Furthermore, the JNK signaling pathway is abnormally active in platinum-resistant ovarian cancer, but platinum drugs can also promote apoptosis of ovarian cancer cells by activating the JNK signaling pathway [35–37]. Because the effect of the JNK signaling pathway in carcinoma is extremely complex but crucial, it may, therefore, be a potential target for molecular cancer treatment therapy.

## JNK and ovarian tumor: role in the death

Cell death is important for maintaining the intracellular homeostasis environment and is critically regulated by the signaling pathway. The ability to increase the number of tumor cells depends not only on the rate of cell proliferation, but also on the rate of cell depletion [38]. Once the signal-regulated pathways of apoptosis, autophagy and other cell functions become abnormal, this may weaken the cell depletion rate for tumorigenesis so as to accelerate the development of cancer. Cell death includes necrosis and apoptosis. Cell apoptosis, a programmed death, is characterized by changes in the nucleus and forming apoptosis body. The death receptor pathway, mitochondrial pathway and endoplasmic reticulum pathway can cause cell death, in addition, autophagy can also cause cell apoptosis.

After the discovery of lysosomes, the Greek term autophagy was proposed. Autophagy can be divided into auto and phagy according to the root, which refers to the process in which organelles in the cytoplasm are transported to lysosome and degraded [39]. Autophagy is formed by double membrane vesicles and lysosomes, namely the autophagy body, which swallows cell damaged or harmful protein, organelles and complexes and the damaged organelles and protein is degraded by autophagy body. Although autophagy protects both normal and cancer cells from apoptosis, especially in the environment of cytotoxicity, malnutrition and other stimuli, when exceeding a certain limit, autophagy can cause apoptosis of normal and cancer cells [40–42]. Autophagy can generally be divided into at least three major categories: macrophage, micro-autophagy and chaperone-mediated autophagy [43]. Usually autophagy refers to macrophage. Many researches have reported that autophagy plays a dual role, helping in cell survival or inducing cell death, depending on the strength or kinds of stimuli and the type of cell.

The JNK pathway plays an essential role in OC autophagy and cell death. Autophagy mediated by the JNK signaling pathway, not only can help OC cells avoid apoptosis when the tumor cells are in a nutrient-poor or cytotoxicity environment, but also can induce autophagy-mediated cell death. Activating JNK signaling pathway induce cell death of autophagy in OC cell, which through promoting conversion of LC3-I to LC3-II as well as forming autophagosomes, accompanying with inhibition of cell cycle in G1 stage via up-regulating the expression of p27 and p21 [44]. Unfolded protein response (UPR) and inositol-requiring enzyme 1α(IRE1α) are essential to autophagy. The character of the endoplasmic reticulum stress (ER stress) response, caused by nutrient deprivation, hypoxia, mitochondrial dysfunction, chemotherapy and etc., can enable the activation of PERK (double-stranded RNA activated protein kinase-like ER kinase), IRE1α and ATF6 and is also controlled by JNK [42]. ER stress results in the accumulation of incorrect folded proteins in cell, although there are some ways help deal with those proteins such as UPR mediated by three different ER transmembrane receptors, ATF, PERK and IRE1, when these protective responses are not enough to cope with ER stress, cells would go through apoptosis in the end [45]. IRE1α is also an essential regulator of the JNK pathway. UPR autophagy-mediated cell death might rely on influencing the activity of IRE1α-JNK [46]. The chaperone proteins Gadd1533 and GRP78 of ER also play an important role in UPR-induced cell death or survival [46, 47]. When UPR in cells exceed a certain limit, damaged cells will die. This may be due to the activation of JNK/AP-1/Gadd153 so as to inhibit the expression of NF-kappa B or Bcl-2 and mediation of ATF6 and ATF4 in OC cells [46, 48]. Furthermore, it is reported that ER stress could not induce apoptosis in caspase-12 deficient cells [49]. This result might be due to IRE1 needing to gather TRAF2 as well as caspase-12 so as to induce cell apoptosis [49, 50]. The use of drugs related to ER stress or autophagy and silencing of autophagy-related proteins Beclin 1 and LC3 can increase cell survival in OC cells [42].

Low-glucose not only induces cell apoptosis via ER stress /IRE1α/JNK, but also influences the generation of cell ATP resulting in cell death via the activation of ASK1 [51]. ASK1 activation leads to sustaining the activation of JNK [51, 52]. And energy metabolism reprogramming is one of the hallmarks of cancer [38]. Dr. Otto Warburg pointed out that cancer cells mainly rely on glycolysis. Furthermore, due to the low efficiency of glycolysis, a high rate for taking up glucose is essential for cancer cells [53]. Low glucose environments could, therefore, enhance some drugs cytotoxicity such as metformin via the energetic stress producing reactive oxygen species (ROS), influencing the mitochondria membrane potential and other byproducts of damaged cells originating from the process of mitochondrial oxidative phosphorylation [51, 54]. ROS-mediated cell apoptosis might, therefore, activate the ASK1/MKK4/7/JNK/mitochondrial signaling pathway [36]. AIF induces apoptosis through the mitochondrial pathway from mitochondrial translocation to the nucleus [55]. Furthermore, Bcl-2 expression decreases, Bax/Bak expression increases, while caspase 3/8/9 does not significantly change [56–58]. SP600125, a specific inhibitor of JNK/SAPK, prevents the expression of Bcl-2, increases the expression of Bax and AIF nuclear translocation, so as to reduce cell apoptosis [59]. Activation transcription factor 2(ATF-2), a component of AP-1, activates the JNK/SAPK pathway-mediated apoptosis of cells via increasing the trimethylation of histone H3K9 associated with the AP-1 binding region interacting with the Bcl-2 promoter [58]. Inhibition of the Akt signaling pathway and c-FLIPL activation can induce the apoptosis of OC cells through up-regulation of p-JNK and subsequent activation of caspase-3 [60]. However, there are also conflicting experimental results suggesting that inhibiting JNK activity may promote cancer cell death. SP600458 can significantly enhance the death of OC cells caused by PARP1 protein cleavage and caspase-3 by disrupting MMP in nilotinib [61]. This may be due to the different subtypes of JNK that play a role. It has been pointed out that WBZ_4, a new inhibitor of JNK1, or targeting A2780CP20, SKOV3 and HEYA8 with JNK1 siRNA, can significantly inhibit the proliferation of OC cells [62].

Furthermore, a clinical trial of ovarian cancer (NCT01015118) indicated that, Nintedanib, which is a triple vascular kinase inhibitor of the VEGF receptor, a platelet-derived growth factor receptor and a fibroblast growth factor receptor, combined with carboplatin and paclitaxel can significantly improve progression-free survival in patients with advanced ovarian cancer [63, 64]. Nintedanib can up-regulate the expression of pulmonary surfactant protein D in A549 cells through the JNK/AP-1 pathway, and alleviate lung fibrosis, however, this does not affect cell proliferation [65] but it can inhibit the proliferation of prostate cancer cells [66]. Concerning ovarian cancer, it is clear that further research is needed to demonstrate how Nintedanib affects the development of ovarian cancer through JNK signaling pathway.

**Fig. 1:**
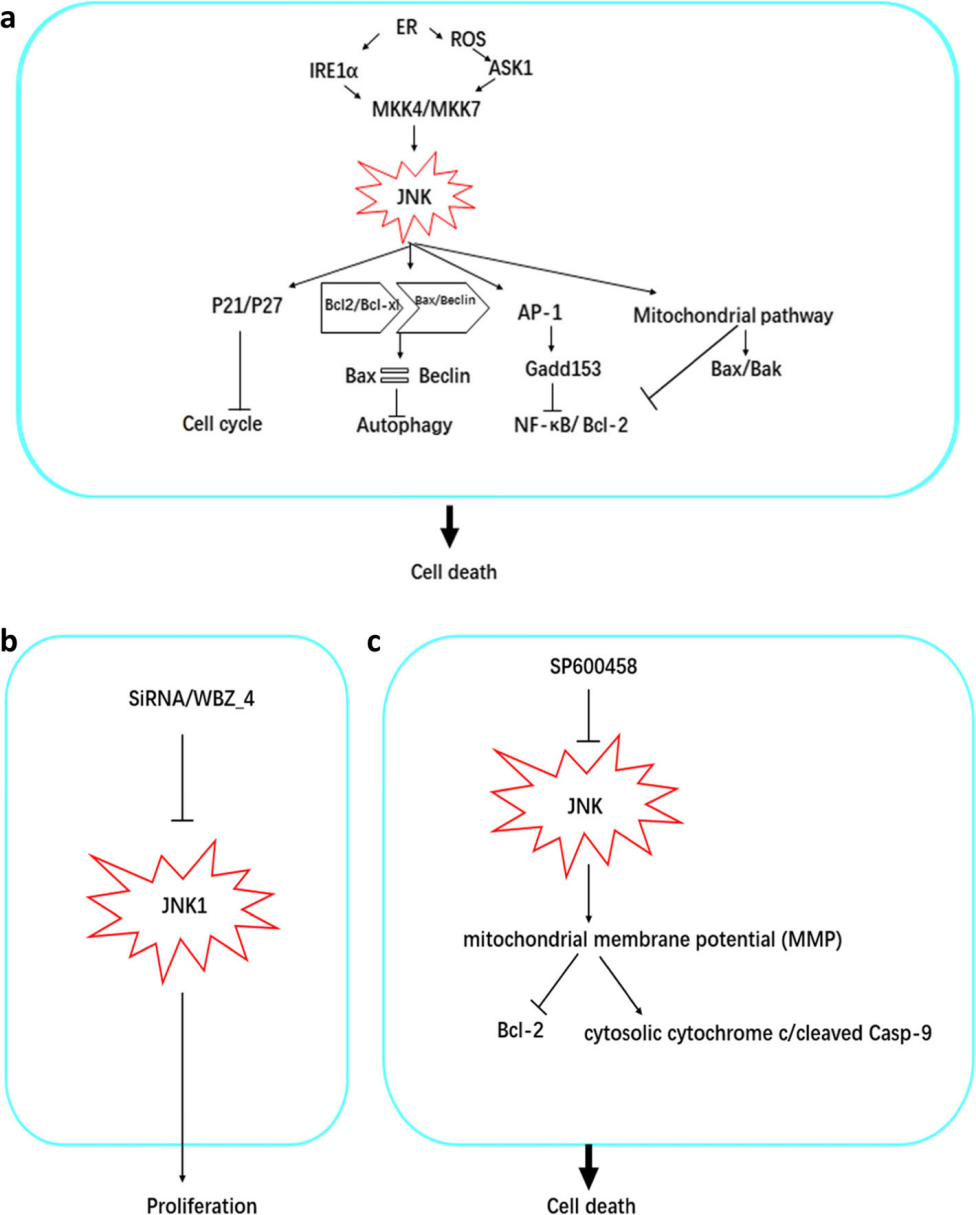
A schematic diagram of the JNK signaling pathway promoting cell death in ovarian cancer. **a** Endoplasmic reticulum stress (ER), reactive oxygen species (ROS), and the Akt signaling pathway can regulate IER1 alpha, UPR and A SK1, thereby activating MKKK4/7 to regulate JNK signaling pathway activity, leading to autophagic cell death, mitochondrial pathway-mediated cell death and AP-1-induced apoptosis. Death-related antibodies mediate cell death. In addition, the JNK signaling pathway can regulate the expression of P27/P21 and cause cell cycle arrest. **b** SiRNA and the JNK related inhibitor WBZ_4, inhibit JNK1 expression and cell proliferation. **c** The JNK inhibitor SP600458 affects the JNK signaling pathway and destroys MMP, thereby enhancing the expression of PARP1 and Caspase-3 and, therefore, promoting cell death

At present, there are only a few reports on JNK inhibitors in the treatment of ovarian cancer; this may be due to the dual role of the JNK signaling pathway, discussed above, in promoting ovarian cancer apoptosis. For instance, mice lacking only one mutant of jnk1/2/3 and either a mutant of jnk1/jnk3 or jnk2/jnk3 can survive normally, but lacking JNK1 and jnk2 at the same time leads to the death of mice embryos and a very serious abnormal apoptosis in their brain cells [67]. In addition, the phosphorylation of JNK1, JNK2 and c-jun is necessary for ultraviolet-induced apoptosis, but JNK2 activated by ER stress can promote the survival of cells [36, 68]. The mechanism of JNK-induced cell death in ovarian cancer is depicted in Fig. 1.

## The function of JNK in the regulation ovarian cancer cell growth

Normal cells need to be regulated by growth signals to change from a stationary state to a proliferative state. Without the stimulation of such signals, no normal cells will proliferate [69]. However, for cancer cells, the proliferation behavior relying on endogenous growth signals is greatly reduced, probably because cancer cells mainly rely on the regulation of carcinogenic genes and their own growth signals. In addition to self-sufficient growth signals and gene mutations, autophagy may also promote tumorigenesis and development by avoiding cell death through internal regulation.

The type of high level serous ovarian cancer with highly aggressive ability is the cause of most, 70–80% [70], of ovarian cancer deaths. Furthermore, this pathological type is the most common type of ovarian cancer. TP53 mutation may induce cell loss through the normal function of anti-cancer, which covers or masks the role of the p53 in normal cell. Carcinomas with TP53 mutations generally have the characteristics of high aggressiveness, invasiveness, and poor-differentiation. According to the report, the frequency of mutation in ovarian cancer ranges from about 50 to 100% [70, 71], such a high rate is attributed to the different histological types of ovarian cancers having different frequencies of mutation. It shows that the frequency of TP53 mutation is rare in low level serous cancer or borderline carcinomas, whereas TP53 mutation is very common in high level serous ovarian cancer, even reaching 100% in some samples [71, 72]. The mutation of TP53 can weaken JNK/ROS/TP53 signaling pathway-mediating cell apoptosis. The tumorigenic effect of JNK is not only weakened by inhibition activity, but also can be promoted by JNK itself. Furthermore, there are other gene mutations in ovarian tumors such as KRAS, BRAF, and PTEN, which induce the activation of the JNK signaling pathway to promote growth of cancer cells [62]. In addition, the mutation of RAS can also contribute to the growth of ovarian cancer. RAS family genes including HRAS, KRAS, NRAS are the most common genes to undergo mutations in tumors. Ras^v12^ mutations interact with JNK-Drosophila TGF-βactivated kinase 1(dTAK1) signaling mediating the growth of tumors, contributing to their proliferation out of control behavior [73]. This might be another reason for inducing the rapid growth of ovarian cells but further experiments need to be done to confirm this. As an important target molecule downstream of JNK, the expression level of c-jun in ovarian cancer tissues is high and is correlated with malignancy [34]. Therefore, inhibiting the signaling pathway of JNK1/c-jun should result in the ability of cellular proliferation and invasion to be significantly decreased [74, 75]. Tumor necrosis factor-alpha-mediated apoptosis can induce transient activation of JNK and regulate NF-kappa B to protect cells from apoptosis [68]. Furthermore, it is positively correlated with alpha 1,2-fucosyltransferase 1/2(FUT1/2) as well as Lewis y, a sugar of type II. The combination of c-jun and the promoter of FUT1 induce the proliferation of ovarian cancer cells and the complexity of Lewis y/ FUT1 might be regulated by TGF-β1 [76, 77]. It has been pointed out that c-Fos can enhance the proliferation of ovarian cancer cells induced by TGF-β1/c-jun [77]. Knocking out c-Fos can down-regulate the expression of cyclin D1 and CD44 as well as inhibit the proliferation and invasion of ovarian cancer cells [77, 78]. Aging human peritoneal mesothelial cells may promote the proliferation and migration of ovarian cancer cells through AP-1/c-jun, IL-6, TGF-βas as well as the phenotypes secreted by tumors, such as angiogenic agents including vascular growth factor and CXCL1 [79].

Moreover, there is also evidence to suggest that the JNK signaling pathway has the function of promoting cancer cell survival in the regulation of cancer cell autophagy and growth [68]. Autophagy can not only induce tumor cell apoptosis as we discussed above, but also induce tumorigenesis and development via the JNK signaling pathway [68, 80]. Many researches have reported that autophagy helps cell survival and proliferation, and in many contexts such as ER stress, chemotherapy, and other diseases can also do so via activating the JNK signal pathway when stimulated for a short time [68, 81]. Different JNK subtypes may have different functions. ER stress can instantaneously activate JNK2 to up-regulate BIP, inhibit cell apoptosis and prevent cell death caused by ER stress, and down-regulate the expression of CHOP, as for transcription factors of cell death and cell apoptosis [68]. Bcl-2, downstream molecular of JNK signaling pathway during the autophagy. Aslo, the JNK signaling pathway, activated by IRE1α, may regulate Bcl-2 phosphorylation of Ser70 via JNK1 not JNK2, thus promoting cell survival [40, 82]. Inhibiting the activity of JNK led to the impair of cancer cell development [83]. TP53 has a negative regulation of Bcl-2, inducing the alteration of the pro-apoptosis Bax/Bak, which initiates cell apoptosis [84]. Note that, Ovarian cancer, especially high level serous ovarian cancer, has a high frequency mutation of TP53 [71]. Inhibiting the activation of Bcl-2 contributes to cell apoptosis, which suggests that the JNK1-Bcl-2 signaling pathway is crucial for the autophagy helping cell survival. There is, however, another explanation that JNK mediates the separation of Beclin1 and Bcl-2/Bcl-XL combinations and induces autophagy. Instantaneous activation of JNK1 can also induce cell survival, but long-term activation can lead to cell apoptosis [85]. When cells are in a hungry state, it is JNK1 rather than JNK2 or JNK3 that mediates the phosphorylation of Bcl-2 so that Beclin1 dissociates from the combinations and stimulates autophagy [40]. Although autophagy-mediating evading apoptosis can help cancer cells maintain a proliferating cell base through intracellular regulation mechanism. Self-regulation mechanism of autophagy may be one of the important reasons leading to drug resistance and recurrence of cancer.

**Fig. 2:**
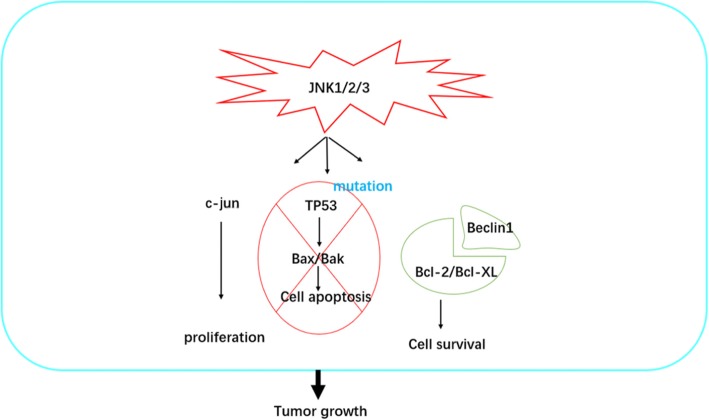
A schematic diagram of the JNK signaling pathway promoting tumor growth in ovarian cancer. C-Jun promotes the proliferation of tumors; JNK-mediated p53 pathway can induce apoptosis, but the mutation of p53 in ovarian cancer can prevent the death of tumors; in addition, JNK signaling pathway

To sum up, the discussion shows that autophagy plays a positive role in the regulation of tumor cell survival via JNK signaling, and gene mutation also promotes tumor growth via activating the JNK pathway. JNK1 induces phosphorylation of Bcl-2, dependent on the length of stimuli, which promotes cells survival. Further research is, therefore, essential to clearly explain the mechanism involved in JNK signaling preventing cell death. Although there are a number of studies in other cancer research areas concerning the promotion of cell survival and growth via the JNK pathway, there are very few in the realm of ovarian cancer and those few only show that inhibiting the JNK pathway impairs the growth of ovarian cancer without demonstrating the underlying mechanism. A potential mechanism, as discussed, for the function in JNK signaling pathway-mediated tumor cell survival and growth is displayed in Fig. 2.

## The role of JNK pathway in chemotherapy resistance

The chemotherapy resistance development of cancer cells is one of the important reasons for impairing the effect of drugs, and may be due to an abnormal activation pathway. Such a resistance phenomenon of tumor cells generally occurs after the initial chemotherapy is given, and in the later stage, drug resistance, aggravation, followed by recurrence may happen in a process similar to Darwin’s natural selection rules, the survival of the fittest. As the drug time increases, the resistance of tumor cells will gradually increase. Chemotherapy kills most ovarian cancer cells, but some cancer stem cells with unique hereditary characteristics survive and represent a hidden danger for future recurrence and drug resistance [86]. In addition, the body’s immune system is also a very important accomplice for the recurrence of ovarian cancer, in that through the use of immune cell recognition functions by tumor cells, they can modify their own immunogenicity, namely cancer immune editing [87, 88]. For an instance, cisplatin, a first line chemotherapy drug for the treatment of advanced ovarian tumors, induces the rate of cancer cell death up to more than 50% but the subsequent treatment effect is decreased owing to drug resistance or recurrence after a long time of chemotherapy exposure [89].

Therefore, in order to look for potential therapeutic targets of chemotherapy resistance to improve the effect of treatment, many researches focused on studying the mechanism of chemotherapy drug resistance. The activation of the JNK signaling pathway plays an important role in promoting drug resistance in cancer [90, 91]. Furthermore, there are also many researches reporting that c-jun is overexpressed in cisplatin resistance in ovarian cancer tissue [34]. The negative expression of c-jun mutation can enhance the sensitivity of the human ovarian cancer cells Caov-3 and A2780 to cisplatin [92]. JNK activity is higher in cisplatin or paclitaxel-resistant human ovarian cancer cell lines and has the positive correlation with drug resistance. In addition, the high expression of active JNK is reported to have a close relationship with stage III and stage IV compared with stage I and stage II, which also was negatively correlated with the survival rate of patients with ovarian cancer [92]. Activation of JNK may play a key role in the DNA repair mechanism due to cisplatin therapy. When cisplatin and SP600125 were combined to treat ovarian cancer cells, the anti-cancer effect was improved compared with cisplatin alone. Compared with the combination of paclitaxel and SP600125, the anti-cancer effect of paclitaxel is weakened, because the death signal caused by paclitaxel is related to the activity of JNK. However, pretreatment of ovarian cancer cells with SP600125 can improve the anti-cancer effects of cisplatin and paclitaxel [92]. Platinum-based drugs increase the level of ROS via the mitochondrial apoptosis signaling pathway. ROS, however, damages the structure of DNA, Protein and other molecular integrity. Note that, sirtuin6, phosphorylated at serine 10 by the JNK pathway, modifies Kap1 so as to promote the silence of L1 retrotransposons [93], and SIRT6 also mediating single adenosine diphosphate ribosylation of PARP1 increases the activity of PARP1 poly adenosine diphosphate ribosylation and promotes DNA double strand break repair [93]. It may also be related to JNK-induced autophagy or gene mutation of tumors themselves helping cells survive. After all, JNK can induce both autophagy and apoptosis by regulating the interaction between Beclin1-Bcl2 complex and Bcl2-Bax [94, 95]. Activation of JNK leads to the destruction of the Bax-Bcl2 complex, which increases the expression of Bax promoting cell apoptosis [96]. JNK-induced autophagy depends on the dissociation of Beclin1, which comes from the Beclin1-Bcl2 complex by phosphorylation of Bcl2. The production of ROS results in mitochondrial damage. There is a small positive regulatory loop between activating apoptosis; ROS, JNK and p53, that is, ROS can activate the JNK signaling pathway. Separation of the p53-MDM2 complex can lead to ROS accumulation, while JNK can activate the downstream gene p53 [95]. The ROS/JNK signaling pathway can promote rapid cell apoptosis through p53; however, mutation of p53 in some ovarian cancer tissue may avoid ROS/JNK/p53 signaling pathway mediated rapid apoptosis of cells.

Micro RNAs (miRNA), having the length of about 21 nucleotieds, do not participate in coding protein directly; these are also be called non-coding RNAs. MiRNAs regulate the expression of gene transcription and also have an association with chemotherapy drug resistance as well as the progression and oncogenesis of cancer. MiR-21, overexpressed highly in many cancers such as breast cancer and ovarian cancer, induces the resistance of cisplatin or paclitaxel [75, 97]. The promoter region of pri-miR-21 interacts with p-c-jun specifically and c-jun is activated by JNK1 not JNK2 or JNK3 in the chemotherapy resistance of ovarian tumor cells [75]. MiR-21 inhibits the expression of programmed cell death 4 (PDCD4), which is directly related to poor prognosis of ovarian tumor patients [98]. Besides, miR-21 also regulates the expression of hypoxia inducible factor-1α influencing tumor cell metabolism [99]. Fra-1, a member of the Fos family, form dimer AP-1 with the Jun family. AP-1 regulates miR-134 so as to augment the function of the JNK signaling pathway mediating chemotherapy insensitivity of ovarian tumor cells [100]. However, not all miRNAs promote the development of tumor chemotherapy resistance. For example, miR-139-5p can interact with c-jun by binding site 3′ UTR of mRNA, which interferes with the combination of c-jun and ATF2 induced by cisplatin and also inhibits the expression of Bcl-xl in cisplatin resistance cells [101].

**Fig. 3:**
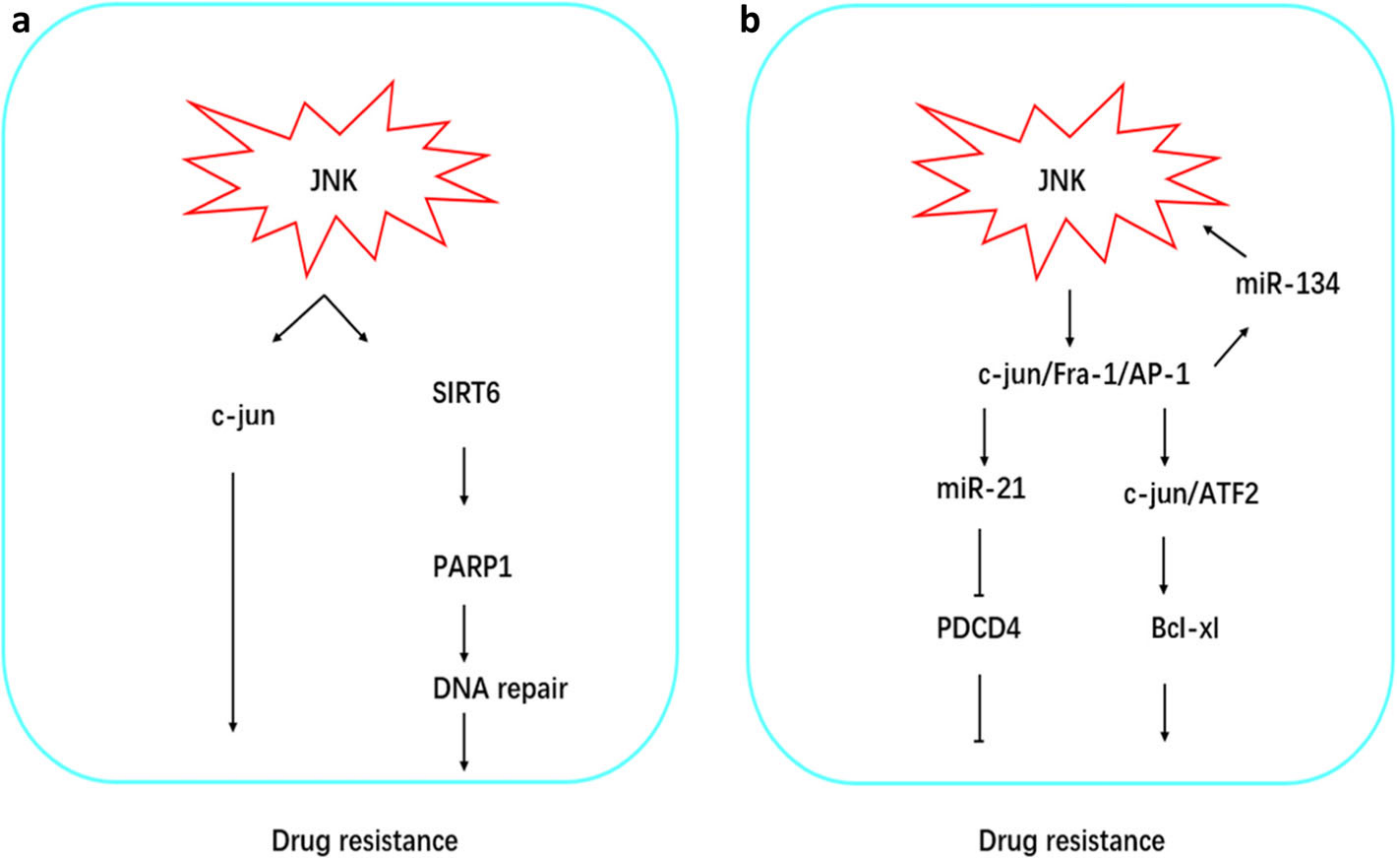
JNK signaling pathway mediated drug resistance. **a** NK signaling pathway mediated by the JNK signaling pathway in ovarian cancer can repair DNA damage through SIRT6/PARP1 or reduce the sensitivity of ovarian cancer tissues to chemotherapy by c-Jun. **b** Drug resistance mediated by JNK signaling pathway also involves microRNA. MiR-21 inhibits the expression of PDCD4 and promotes resistance, or the interaction between miR-134 and JNK signaling pathway promotes the activation of c-jun/ATF2 and promotes drug resistance

These researches suggest that the JNK signaling pathway plays an essential role in chemotherapy resistance of ovarian cancer cells, although its functions are contradictory in tumors. Autophagy mediated by the JNK signaling pathway may provide resistance to chemotherapeutic drugs, leading to drug resistance in the later stage of relapse. Mutations in the P53 gene may inhibit apoptosis induced by ROS/JNK/p53, although the JNK pathway may induce apoptosis in a caspase-dependent manner. Studies have shown that the staggered use of JNK inhibitors and the chemotherapeutic drugs platinum or paclitaxel in turn can increase chemotherapeutic sensitivity without increasing side effects. The mechanism of JNK-mediated chemotherapeutic drug resistance discussed above is shown in Fig. 3. It is clear that JNK-mediated drug resistance in ovarian cancer chemotherapy is a difficult problem that needs to be solved as soon as possible.

## Conclusion

It is clear that understanding dual role of the JNK signaling pathway, that can both promote the development of tumorigenesis and also inhibit the progress of tumors, the so called seesaw role, is very important in the study of cancer. The immune escape mechanism of tumors may also be involved in JNK-mediated tumor cell survival, such as interleukin and tumor necrosis factor. Although many studies have provided detailed evidence for JNK to promote the survival of cancer cells and inhibit the development of cancer, many problems remain unsolved, including the following: (1) JNK signaling pathway mediates death in the early stage and induces drug resistance in the later stage. Besides the reason of activation time and phosphorylation SIRT6 so as to repair DNA breaks, how it specifically affects JNK signaling pathway has not been confirmed. And vivo experiments and clinical studies need to be improved. (2) JNK1 can promote cell proliferation, and autophagy induced by external stimulation of the JNK signaling pathway and can also help cancer cells avoid apoptosis induced by drugs. But under what stress conditions these external stimuli and chemotherapeutics cause autophagic apoptosis rather than autophagy. (3) At present, the evidence of many oncology experiments related to the JNK signaling pathway comes from cell experiments, especially on ovarian cancer cells. The study of mouse models and clinical trials will help to improve our understanding of the function of the JNK signaling pathway in tumors. Because the occurrence and development of cancer is affected by the overall internal environment of the body, not a single factor.

The JNK signaling pathway regulates both cell death and survival. It is, therefore, important to study the role of the JNK signaling pathway in each of these conflicting situations and the mechanisms involved in “see-sawing” between each other. In the future, it is necessary to determine the specific relationship between JNK structure and function. In view of this though, we firmly believe that in the near future, defining the specific mechanism of JNK in the process of tumorigenesis and development, and targeting the JNK signaling pathway in cancer treatment-related drugs will provide tremendous help to spearhead a breakthrough in anti-cancer treatment.

## Data Availability

Not applicable

## Abbreviations

ASK1: Apoptosis signal regulation kinase 1
ER stress: Endoplasmic reticulum stress
JNK: Jun N-terminal kinase
MAPK: Mitogen-activated protein kinases
OC: Ovarian carcinoma
ROS: reactive oxygen species
